# Impact of a Mobile App (LoAD Calc) on the Calculation of Maximum Safe Doses of Local Anesthetics: Protocol for a Randomized Controlled Trial

**DOI:** 10.2196/53679

**Published:** 2024-01-03

**Authors:** Pietro Elias Fubini, Georges Louis Savoldelli, Tal Sara Beckmann, Caroline Flora Samer, Mélanie Suppan

**Affiliations:** 1 Division of Anesthesiology Department of Anesthesiology, Clinical Pharmacology, Intensive Care and Emergency Medicine Geneva University Hospitals and Faculty of Medicine Geneva Switzerland; 2 Clinical Pharmacology and Toxicology Division Department of Anesthesiology, Clinical Pharmacology, Intensive Care and Emergency Medicine Geneva University Hospitals and Faculty of Medicine Geneva Switzerland

**Keywords:** dose calculation, drug safety, information systems research, local anesthetic systemic toxicity, local anesthetics, mobile health app, study protocol, toxicity

## Abstract

**Background:**

Local anesthetics (LAs) are regularly used to alleviate pain during medical or surgical procedures. Their use is generally considered safe, but exceeding the maximum recommended doses can lead to LA systemic toxicity, a rare but potentially lethal complication. Determining maximum safe doses is therefore mandatory before performing local anesthesia, but rules are often unclear and the factors affecting dose calculation are numerous. Mobile health apps have been shown to help clinical decision-making, but most currently available apps present significant limitations. The Local Anesthetics Dose Calculator (LoAD Calc) app was designed to overcome these limitations by taking all relevant parameters into account. Before deploying this app in a clinical setting, it should be tested to determine its effectiveness and whether clinicians would be willing to use it.

**Objective:**

The primary objective will be to evaluate the effectiveness of the LoAD Calc app through written simulated cases. The secondary objective will be to determine whether physicians find this app easier, faster, and safer than the methods they generally use.

**Methods:**

We describe a parallel-group randomized controlled trial protocol. Anesthesiologists working at the Geneva University Hospitals will be invited to participate. Participants will be asked to compute the maximum dose of LA in 10 simulated clinical cases using 3 different LAs. The maximum safe dose will be determined manually using the same calculation rules that were used to develop LoAD Calc, without using the app itself. An overdose will be considered any dose higher than the correct dose, rounded to the superior integer, while an underdose will be defined as the optimal calculated dose minus 20%, rounded to the inferior integer. Randomization will be stratified according to current position (resident vs registrar). The participants allocated to the LoAD Calc (experimental) group will use the LoAD Calc app to compute the maximum safe LA doses. Those allocated to the control group will be asked to use the method they generally use. The primary outcome will be the overall overdose rate. Secondary outcomes will include the overdose rate according to ideal and actual body weight and to each specific LA, the overall underdose rate, and the time taken to complete these calculations. The app’s usability will also be assessed.

**Results:**

A sample size of 46 participants will be needed to detect a difference of 10% with a power of 90%. Thus, a target of 50 participants was set to allow for attrition and exclusion criteria. We expect recruitment to begin during the winter of 2023, data analysis in the spring of 2024, and results by the end of 2024.

**Conclusions:**

This study should determine whether LoAD Calc, a mobile health app designed to compute maximum safe LA doses, is safer and more efficient than traditional LA calculation methods.

**International Registered Report Identifier (IRRID):**

PRR1-10.2196/53679

## Introduction

### Background

Local anesthetics (LAs) are used daily by physicians to perform minor procedures. While the doses they use are generally limited, anesthesiologists often use higher doses to perform regional anesthesia techniques [1]. While the advantages of such techniques are undeniable, using high LA doses increases the risk of local anesthetic systemic toxicity (LAST), a potentially lethal complication associated with the use of these agents [2]. The actual incidence of LAST is unknown since most minor symptoms are not specific and because LAST awareness varies considerably between practitioners [3,4]. The incidence reported in scientific studies varies from 0.04 to 1.8 per 1000 regional anesthesia procedures but is probably underestimated [5,6]. The main risk factors seem to be inadvertent intravascular injections and inappropriately large doses [7,8].

Prevention of intravascular injection can be achieved by ultrasound guidance and careful aspiration during the procedure, while adequate calculation of the maximum dose of LA before administration is the best way to avoid incorrect doses.

Although different guidelines have been created to help clinicians calculate the maximum safe LA doses, quickly and reliably determining such doses often proves difficult in clinical practice [9]. Many anesthesiologists rely on mental calculation (with or without a pen and paper aid), and some use calculators. These methods are, however, often challenging and inaccurate, especially if LA mixtures are used or when patients present multiple comorbidities [10]. More advanced solutions have been developed to support LA dosage calculation, such as the nomogram created by Williams and Walker [11]. The main limitation of this solution is that the nomogram must always be at hand. Moreover, specificities such as ideal body weight (IBW) calculation and adaptation in the case of relevant comorbidities are indicated but not directly integrated into dose determination.

To facilitate the calculation of safe maximum LA doses, a mobile health (mHealth) app, Local Anesthetics Dose Calculator (LoAD Calc), was developed at the Geneva University Hospitals [12]. This app takes all relevant parameters (IBW and actual weight, height, age, medications, and comorbidities) into account and allows the use of a mixture of 2 different LAs. Since smartphones have widely replaced older paging systems and are therefore always at hand, this mHealth app could be an appropriate solution to enable anesthesiologists to efficiently compute safe maximum LA doses.

### Objectives

This study protocol follows the hypothesis that LoAD Calc, an mHealth app designed to help clinicians calculate maximum safe LA doses, is safer and more effective than traditional methods. Thus, the primary objective will be to evaluate the effectiveness of the LoAD Calc app by using it to compute the maximum single doses of LA in written simulated cases. The secondary objective will be to determine whether physicians find this app easier, faster, and safer than the methods they generally use.

## Methods

### Ethical Considerations

A synopsis of the study protocol was presented to the regional ethics committee (Commission Cantonale d’Ethique de la Recherche [CCER]). This committee confirmed that this project does not fall within the scope of the Swiss Federal Act on Research involving Human Beings and issued a “declaration of no objection” (CCER 2022-01577) [13]. This study protocol does not fall within the scope of the Swiss Federal Act on Research involving Human Beings [13]. It will nevertheless be presented to the regional ethics committee to ascertain that no important or relevant ethical consideration was omitted.

Participants will be told that participation is entirely voluntary, that there will be no consequence if they refuse to participate, and that they will be able to withdraw at any time without explanation. All participants will be asked to sign an electronic consent form immediately after logging in. There is no compensation for participation in the study.

### Study Design

This will be a monocentric, parallel-group, randomized controlled trial based on clinical vignettes. The protocol was developed according to the Standard Protocol Items: Recommendations for Interventional Trials (SPIRIT) statement (Multimedia Appendix 1) [14]. Given its design, the investigators will not be blinded as to the intervention. Nevertheless, participants will not be informed that there are 2 different arms and will not be told the exact outcomes studied, even though they will be provided with general information regarding the study. In addition, the data analyst will be blinded as to participant allocation by renaming the groups before sending data for statistical analysis. Randomization will be stratified according to current position (resident vs registrar).

Results will be reported according to the CONSORT-EHEALTH (Consolidated Standards of Reporting Trials of Electronic and Mobile HEalth Applications and onLine TeleHealth) guidelines [15]. Relevant elements of the CHERRIES (Checklist for Reporting Results of Internet E-Surveys) will be included since web-based questionnaires will be used in the course of this study [16].

### Clinical Vignettes

A total of 10 clinical vignettes will be developed for the purpose of this study. These vignettes will describe clinical cases requiring the use of LAs for regional anesthesia. We will include 3 of the most commonly used LAs in these vignettes: lidocaine, levobupivacaine, and ropivacaine. Some vignettes will ask the participant to use LA mixtures, and several will include comorbidities or medications requiring dose adaptations.

For each vignette, 3 authors will be required to determine the maximum dose of LA the simulated patient should receive according to the rules used to develop the LoAD Calc app [12], without using the app. These rules, which are derived from the scientific literature, are summarized in Textbox 1. They were reviewed and approved by clinical pharmacologists and toxicologists [12]. Any disagreement will prompt a review of the vignette. Final vignette approval will only be possible if a consensus can be reached.

**Dosage elements and app rules used to calculate the maximum safe dose of local anesthetics, adapted from Suppan et al [12].**

**Dose limit for a single LA (local anesthetic)**

- Levobupivacaine: 2 mg/kg (maximum 150 mg/dose)
- Lidocaine: 3 mg/kg (maximum 300 mg/dose)
- Ropivacaine: 3 mg/kg (maximum 225 mg/dose)

**Influence of epinephrine on dose limit**

- Levobupivacaine: 3 mg/kg (maximum 150 mg/dose)
- Lidocaine: 7 mg/kg (maximum 400 mg/dose)
- Ropivacaine: 3 mg/kg (maximum 225 mg/dose)

**Determination of calculation weight (CW)**

- Calculation of BMI
- Calculation of ideal body weight (IBW; Devine formula)
- Application of the following algorithm to define CW:
  - weight≤70 kg and BMI<30 and IBW >weight → CW=weight
  - weight≤70 kg and BMI<30 and IBW ≤weight → CW=IBW
  - weight≤70 kg and BMI≥30 → CW=IBW
  - weight>70 kg and IBW>70 → CW=70
  - weight>70 kg and IBW≤70 → CW=IBW

**Dose adaptation depending on health conditions and drugs**

- Conditions
  - Old age (70 years or older)
  - Renal dysfunction (glomerular filtration rate [GFR]<50 mL/minute)
  - Hepatic insufficiency (prothrombin time <50%)
  - Heart failure (left ventricular ejection fraction≤30%)
  - Pregnancy
  - Drugs decreasing LA metabolism
- List of drugs decreasing LA metabolism
  - Major CYP1A2 inhibitors: ciprofloxacin, norfloxacin, and fluvoxamine
  - Major CYP3A inhibitors: azole antifungals, macrolides, calcium channel blockers, HIV antiretroviral therapy, and tyrosine kinase inhibitors
- If 1 condition is present, the calculator reduces the total maximum dose by 20%
- If 2 or more conditions are present, the calculator reduces the total maximum dose by 30%.

**Calculation rule for LA mixtures**

- The app performs the following steps
  - Calculation of maximum safe volume for first LA
  - The user enters which volume of first LA is to be used (0–maximum volume)
  - Calculation of corresponding maximum dose of first LA and determination of percentage of total maximum dose
  - Calculation of maximum dose of second LA based on remaining percentage of total maximum dose
  - Calculation of maximum volume of second LA

### Groups and Randomization

There will be 2 study groups: in the control group, participants will be asked to use the method they usually use in their clinical practice to calculate the maximum safe dose of LA; in the LoAD Calc (experiment) group, participants will be required to use the LoAD Calc app, which will be preinstalled on a standard Geneva University Hospitals (HUG) smartphone (Galaxy XCover 4s; Samsung) running on Android 11.

Stata’s (StataCorp LLC) replicable balanced randomization mechanism will be used to allocate participants to their study group. Textbox 2 contains the code that will be used.

**Randomization code.**

*set obs #N*

*egen arm = seq(), to(2)*

*set seed #S*

*gen random = uniform()*

*sort random*

Wherein “1” will be the control group, and “2” will be the LoAD Calc group.

Since randomization will be stratified according to participant position (either resident or registrar), 2 seeds (#S) will be used (07022023 and 20230207).

A sample size calculation will be used to determine the total number of observations. It will be rounded up to the nearest ten to enhance the study power and take into account attrition and potential exclusions. The stratified number of observations (#N) will be computed according to the proportion of potential participants belonging to both eligible positions (residents vs registrars).

The method used by the participants allocated to the control group to calculate the maximum safe dose of LA will be recorded. There will be no teaching or introductory intervention for any of the participants before the study, and the participants allocated to the LoAD Calc group will therefore discover the app while answering the first vignette.

### Web-Based Study Platform

A specific web-based platform will be developed using the Joomla! 4.3 content management system (Open Source Matters). It will be hosted on a Swiss server (Kreativmedia GmbH) and secured by the RSFirewall 3 (RSJoomla) and AdminTools 7 (Akeeba Ltd) components. To ensure participant anonymity, unique usernames and passwords will be created using Manytools’ web-based password generator [17]. These credentials will then be imported into Stata and allocated to either study group according to the randomization process described above. Finally, this data will be exported to a CSV file, which will be imported into the web-based study platform using the Import Joomla Users component (version 3.4; Lerus Ltd).

Consents, questionnaires, and vignettes will be managed using Shondalai’s Community Surveys 6 and Community Quiz 6 components (Bulasikku Technologies Pvt Ltd). All data will be stored on an encrypted MySQL-compatible database (MariaDB 10, MariaDB Foundation).

### Inclusion and Exclusion Criteria

All resident physicians and registrars working in the HUG anesthesiology department will be eligible for inclusion. The only exclusion criteria will be current or previous use of the LoAD Calc app. This criterion will be assessed by a screening question asked after the completion of all study vignettes.

### Recruitment

The project will first be presented to the head of the anesthesiology department and then to all consultants. After obtaining their agreement, investigators will recruit potential participants directly in the operating room. These residents and fellows in anesthesiology will be informed that the study will last at most 1 hour and that an investigator will replace them in the operating room while they participate. They will be told that participation is entirely voluntary, that there will be no consequence if they refuse to participate, and that they will be able to withdraw at any time without explanation. No incentive other than advancing scientific knowledge will be given to promote participation. They will be given a paper sheet summarizing the information regarding the study and data protection (Multimedia Appendix 2). Those who agree will be scheduled for participation on the same day. Together with the anesthesiology consultant overseeing the operating room, an investigator will organize replacements to avoid any disruption in the operating program. There will be only 1 slot, and therefore, only 1 participant per hour.

### Consent and Study Sequence

Participants will be asked to set their phones to flight mode. This will enable them to access any note, calculator, or app they use to calculate LA doses while avoiding potentially disruptive interruptions. The study itself will take place in a separate, quiet room. There, an investigator will prompt them to pick up a sealed, opaque envelope containing the credentials necessary to log in.

All participants will be asked to sign an electronic consent form immediately after logging in. Those who agree will proceed to a first questionnaire designed to gather demographical data (Textbox 3) and determine whether these participants are currently using LoAD Calc or if they have used this app before (exclusion criterion). After completing this questionnaire, an introductory screen giving information regarding the vignettes they are about to see and specifying the calculation method they are to use (LoAD Calc for the experimental group vs left at the participant’s will for the control group) will be displayed. At this stage, those allocated to the LoAD Calc group will be given the smartphone preinstalled with the LoAD Calc app.

**First questionnaire.**

**Page 1: consent**

- Consent to participate and to data reuse (multiple-choice questions with only 1 acceptable answer)

**Page 2: exclusion criterion**

- Has heard of LoAD Calc (multiple-choice questions with only 1 acceptable answer)^a^
- Has installed LoAD Calc (multiple-choice questions with only 1 acceptable answer; branching logic will be used to avoid displaying irrelevant questions)
- Has used LoAD Calc (multiple-choice questions with only 1 acceptable answer; branching logic will be used to avoid displaying irrelevant questions; answering “yes” to either of those questions will lead to participant exclusion)
- Context of LoAD Calc use (multiple-choice question with only 1 acceptable answer; branching logic will be used to avoid displaying irrelevant questions)
- Has LoAD Calc still installed (multiple-choice question with only 1 acceptable answer; branching logic will be used to avoid displaying irrelevant questions)
- Still uses LoAD Calc (multiple-choice questions with only 1 acceptable answer; branching logic will be used to avoid displaying irrelevant questions; answering “yes” to either of those questions will lead to participant exclusion)

**Page 3: demographics**

- Gender (multiple-choice questions with only 1 acceptable answer)
- Age (free text with regular expression [regex] validation rule)
- Position (multiple-choice questions with only 1 acceptable answer; custom answer accepted)
- Years since graduation (free text with regular expression [regex] validation rule)
- Years of practice in anesthesiology (free text with regular expression [regex] validation rule)
- Specialist diplomas (custom answer accepted; multiple-answer question with more than 1 answer accepted)

After completing the vignettes, the participants allocated to the LoAD Calc group will be asked to complete the French version of the System Usability Scale [18], the translation of which has been validated [19]. Participants allocated to the control group will then be asked which methods they used to calculate LA doses.

Finally, both groups will have to answer a question based on a 10-point Likert scale to assess their confidence as to the method they used to carry out the maximum safe LA dose calculations, from score 1 (absolutely not confident) to score 10 (perfectly confident).

**Figure 1:**
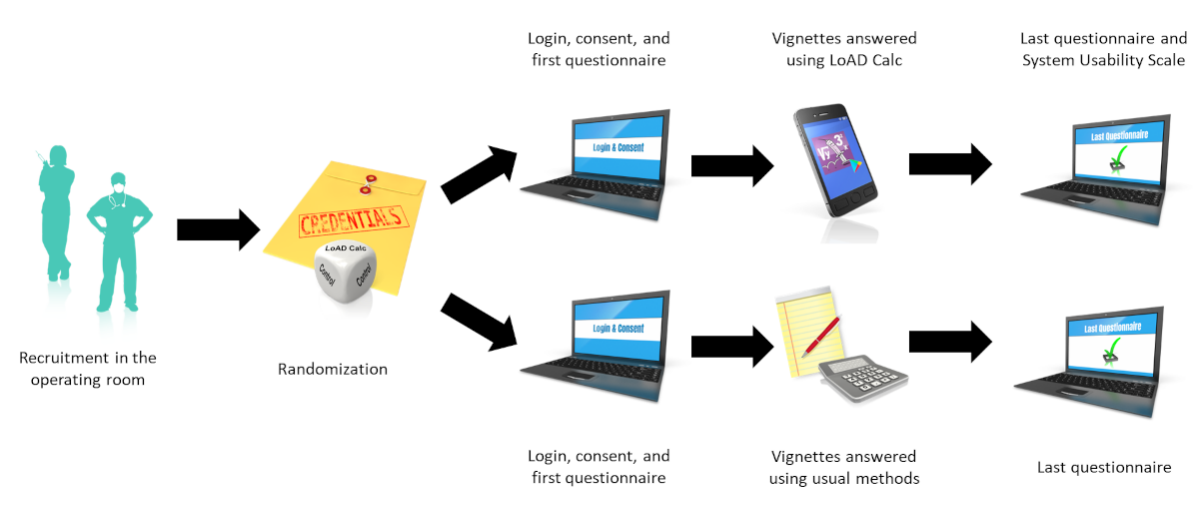
Study sequence. LoAD Calc: Local Anesthetics Dose Calculator.

The whole study sequence is summarized in Figure 1.

### Outcomes

The primary outcome will be the overall overdose rate, according to the method used. To assess this outcome, the maximum acceptable dose in milligrams, or milliliters, must therefore be established. While this might seem straightforward at first sight, it is actually rather complex. Indeed, although maximum safe doses are calculated in milligrams, anesthetists administer a volume of LA (the concentration of which can vary) rather than a quantity of LA. Therefore, even though toxicity is related to the quantity (in milligrams) of LA administered, it is clinically more relevant to determine the maximum volume (in milliliters) of LA that can be used for a particular patient. Consequently, after applying the rules described in Textbox 1, a quantity in milligrams will be obtained. It will then be converted in milliliters according to the concentration of the LA used in the vignette. This volume will then be rounded to the inferior integer. To be less conservative, 1 mm will be added to this calculated volume, and the total will represent the maximum acceptable volume. An overdose will be considered any dose higher than this maximum acceptable volume or than its corresponding LA quantity in milligrams.

The secondary outcomes will be the overall overdose rate, considering the simulated patient’s ideal weight and the simulated patient’s actual weight, the overdose rate according to each LA studied, and the overall underdose rate. An underdose will be defined as the maximum acceptable volume minus 20% (or its corresponding LA quantity in milligrams), rounded to the inferior integer. This is an empirical choice since anesthetic underdose can only be determined clinically [20,21].

Other secondary outcomes will be the time taken to complete these calculations, the app’s usability, and the physicians’ confidence in using the method they were allocated to. The app’s usability will be evaluated using the System Usability Scale [18]. Provided that the statistical assumptions are met, factors associated with a higher probability of overdose or underdose will also be assessed.

### Statistical Analysis

The sample size calculation and all other statistical analyses will be carried out using Stata (version 17.0 or above). The complete data set will be exported by the webmaster, who will give the study groups codenames before sending the curated data set for statistical analysis. Descriptive statistics will be used to present demographical data. Normality will be assessed graphically, and the Kolmogorov-Smirnov test will be used in cases of doubt. Accordingly, all outcomes will then be computed using either parametric or nonparametric tests. The data acquisition mechanisms will ensure that all data are recorded after each stage. Thus, there should not be any missing data, and there shall be no need for imputation. When LA mixtures are used, participants will be told that 1 anesthetic has already been injected and the dose used has been clearly reported. Thus, they will be asked to determine the maximum safe dose for the second local anesthetic. Multivariable regression will be used to determine an association between specific clinical parameters and the probability of overdose or underdose, provided that all required assumptions are met and that the risk of overfitting is adequately limited. Double-sided *P* values (*P*<.05) will be considered significant.

## Results

The 10 vignettes necessary to carry out the study were successfully created, and the maximum safe doses were determined. These vignettes and the doses were checked and approved by all authors. The 10 vignettes, as well as their English translation, were presented to peer reviewers but are not publicly available to avoid any potential bias. They will nevertheless be published along with the results paper.

The sample size calculation was performed using Stata (version 17.0). It showed that 46 participants (23 in each group) would be needed to detect a 10% difference with a power of 90%, taking into account an SD of 10%. In line with the above methods, a total of 50 anesthesiologists should therefore be recruited. Since there are 62 residents and 52 registrars in the HUG Anesthesiology Department, a participation rate of 44% (50/114) will be necessary. This participation rate seems achievable with the aforementioned recruitment procedure. If this rate cannot be achieved, other Swiss University hospitals will be contacted, and similar recruitment procedures will be carried out to obtain the required sample size.

The study platform has been successfully created and tested by all coauthors [22]. The data extraction mechanisms have also been successfully checked.

The recruitment will take place once this study protocol has been reviewed and accepted for publication to allow for any necessary adjustments before study inception. The current version of the protocol is 0.9 (October 10, 2023). The published version will be 1.0.

It should be possible to start recruitment during the winter of 2023. This would allow data analysis to take place in spring 2024, and results should be submitted for publication in an international peer-reviewed journal by the end of the same year.

## Discussion

### Overview

This study should allow us to determine whether LoAD Calc, an mHealth app designed to calculate maximum safe LA doses, is safer and more effective than current clinical practice. Previous studies have shown that mHealth apps can enhance dose calculation and potentially improve safety [23], decrease time to drug delivery [23], and lessen stress [24]. Assessing this latter parameter would not make much sense given the design of this study but could prove interesting in future high-fidelity or field trials.

Other solutions have already been proposed for the calculation of the maximum safe LA dose but present significant drawbacks. Some of them, such as the nomogram created by Williams and Walker [11], do not depend on technological devices. This nomogram, which represents a rapid and calculation-free way, must, however, always be within reach. In addition, IBW must first be determined, and there is no dose adaptation based on health conditions or drug interactions. Computer-based solutions and mobile apps have also been created, such as MDCalc Local Anesthetic Dosing Calculator [25], The Podiatry Institute’s LA Toxic Dose Calculator [26], and SafeLocal by Johns Hopkins Digital [27]. All these solutions lack key elements and do not consider either IBW, comorbidities, or medications. Most allow invalid data to be entered or suggest doses exceeding the maximum safe dose, thereby presenting potential safety issues.

No study can be devoid of limitations, and the one planned according to this protocol is no exception. The first foreseeable limitation is that the LoAD Calc app will be compared to many different methods of LA dose calculation, thereby preventing us from directly comparing this app to a specific method. However, there is no gold standard to calculate the maximum safe LA doses, and the design of the proposed study can be considered pragmatic. Another limitation is that the maximum safe doses will be calculated using the same scientifically grounded rules that were used to develop LoAD Calc [12]. However, some of the calculation rules used by the app are not supported by strong scientific evidence, and there is no gold standard for comprehensive, safe calculation of maximum LA doses. Finally, the results obtained through this study will only apply to the single-dose administration of a limited number of LAs or of LA mixtures. This will not affect the validity of the study’s results nor compromise the use of the app since the 3 LAs selected (levobupivacaine, lidocaine, and ropivacaine) are commonly used in clinical practice. Nevertheless, further app developments will be needed to take other LAs and repeated doses into account. Since some LAs, such as lidocaine, are also safe for intravenous use, future versions of the app should enable practitioners to select different injection sites and routes.

### Conclusions

Following this protocol should enable us to determine whether LoAD Calc, a mHealth app designed to calculate the maximum safe doses of LA, is both safe and effective. If this hypothesis proves to be true, clinical trials could be considered, and further outcomes, such as the impact of LoAD Calc on cognitive load and physiologic stress, could be considered.

## Appendix

Multimedia Appendix 1 SPIRIT checklist.

Multimedia Appendix 2 Study information sheet.

## Abbreviations

CCER: Commission Cantonale d’Ethique de la Recherche
CHERRIES: Checklist for Reporting Results of Internet E-Surveys
CONSORT-EHEALTH: Consolidated Standards of Reporting Trials of Electronic and Mobile HEalth Applications and onLine TeleHealth
HUG: Geneva University Hospitals
IBW: ideal body weight
LA: local anesthetic
LAST: local anesthetic systemic toxicity
LoAD Calc: Local Anesthetics Dose Calculator
mHealth: mobile health
SPIRIT: Standard Protocol Items: Recommendations for Interventional Trials

## Data Availability

The data sets generated during and/or analyzed during this study will be available in an open access repository.
