# Supplementary material for: Impact of a Mobile App (LoAD Calc) on the Calculation of Maximum Safe Doses of Local Anesthetics: Protocol for a Randomized Controlled Trial
Source: JMIR Res Protoc. 2024 Jan 3;13:e53679. doi: 10.2196/53679 (PMC10794956; doi:10.2196/53679)
Supplement: Multimedia Appendix 1 [file resprot_v13i1e53679_app1.pdf]

Section/item	Item	Description	Protocol
Administrative information			
Title	1	Descriptive title identifying the study design, population, interventions, and, if applicable, trial acronym	Title written according to JMIR guidelines (https://support.jmir.org/hc/en-us/articles/115002943791-What-are-JMIR-s-guidelines-for-article-titles-)
Trial registration	2a	Trial identifier and registry name. If not yet registered, name of intended registry	An IRRID should be assigned if the protocol is accepted for publication
	2b	All items from the World Health Organization Trial Registration Data Set	N/A (see 2a)
Protocol version	3	Date and version identifier	Under the results section: *the current version of the protocol is 0.9 (15.10.2023). The published version will be 1.0.*
Funding	4	Sources and types of financial, material, and other support	Under statements: "This research received no external funding."
Roles and responsibilities	5a	Names, affiliations, and roles of protocol contributors	Acknowledged on the title page and under "CRediT Author Statement"
	5b	Name and contact information for the trial sponsor	Acknowledged as corresponding author
	5c	Role of study sponsor and funders, if any, in study design; collection, management, analysis, and interpretation of data; writing of the report; and the decision to submit the report for publication, including whether they will have ultimate authority over any of these activities	Reported under "CRediT Author Statement" and "Conflicts of Interest"
	5d	Composition, roles, and responsibilities of the coordinating centre, steering committee, endpoint adjudication committee, data management team, and other individuals or groups overseeing the trial, if applicable (see Item 21a for data monitoring committee)	Not applicable
Introduction			
Background and rationale	6a	Description of research question and justification for undertaking the trial, including summary of relevant studies (published and unpublished) examining benefits and harms for each intervention	OK - under Introduction/Background
	6b	Explanation for choice of comparators	OK - under Introduction/Background and Introduction/Objectives
Objectives	7	Specific objectives or hypotheses	OK - under Introduction/Objectives
Trial design	8	Description of trial design including type of trial (eg, parallel group, crossover, factorial, single group), allocation ratio, and framework (eg, superiority, equivalence, noninferiority, exploratory)	OK - Under Methods/Study Design
Methods: Participants, interventions, and outcomes			
Study setting	9	Description of study settings (eg, community clinic, academic hospital) and list of countries where data will be collected. Reference to where list of study sites can be obtained	OK - Under Methods/Inclusion and exclusion criteria
Eligibility criteria	10	Inclusion and exclusion criteria for participants. If applicable, eligibility criteria for study centres and individuals who will perform the interventions (eg, surgeons, psychotherapists)	OK - Under Methods/Inclusion and exclusion criteria
Interventions	11a	Interventions for each group with sufficient detail to allow replication, including how and when they will be administered	OK - Under Methods/Consent and study sequence
	11b	Criteria for discontinuing or modifying allocated interventions for a given trial participant (eg, drug dose change in response to harms, participant request, or improving/worsening disease)	Not applicable ("single shot" study)
	11c	Strategies to improve adherence to intervention protocols, and any procedures for monitoring adherence (eg, drug tablet return, laboratory tests)	Not applicable ("single shot" study)
	11d	Relevant concomitant care and interventions that are permitted or prohibited during the trial	Not applicable ("single shot" study)
Outcomes	12	Primary, secondary, and other outcomes, including the specific measurement variable (eg, systolic blood pressure), analysis metric (eg, change from baseline, final value, time to event), method of aggregation (eg, median, proportion), and time point for each outcome. Explanation of the clinical relevance of chosen efficacy and harm outcomes is strongly recommended	OK - Under Methods/Outcomes

Participant timeline	13	Time schedule of enrolment, interventions (including any run-ins and washouts), assessments, and visits for participants. A schematic diagram is highly recommended (see Figure)	OK - Under Methods/Recruitment
Sample size	14	Estimated number of participants needed to achieve study objectives and how it was determined, including clinical and statistical assumptions supporting any sample size calculations	OK - Under Methods/Statistical Analysis and Results
Recruitment	15	Strategies for achieving adequate participant enrolment to reach target sample size	OK - Under Results
Methods: Assignment of interventions (for controlled trials) Allocation:			
Sequence generation	16a	Method of generating the allocation sequence (eg, computer-generated random numbers), and list of any factors for stratification. To reduce predictability of a random sequence, details of any planned restriction (eg, blocking) should be provided in a separate document that is unavailable to those who enrol participants or assign interventions	OK - Under Methods/Groups and randomization
Allocation concealment mechanism	16b	Mechanism of implementing the allocation sequence (eg, central telephone; sequentially numbered, opaque, sealed envelopes), describing any steps to conceal the sequence until interventions are assigned	OK - Under Methods/Groups and randomization
Implementation	16c	Who will generate the allocation sequence, who will enrol participants, and who will assign participants to interventions	OK - Under Methods/Groups and randomization and Methods/Consent and study sequence
Blinding (masking)	17a	Who will be blinded after assignment to interventions (eg, trial participants, care providers, outcome assessors, data analysts), and how	OK - Under Methods/Study Design
	17b	If blinded, circumstances under which unblinding is permissible, and procedure for revealing a participant's allocated intervention during the trial	Not applicable ("single shot" study with no impact on participants' health)
Methods: Data collection, management, and analysis			
Data collection methods	18a	Plans for assessment and collection of outcome, baseline, and other trial data, including any related processes to promote data quality (eg, duplicate measurements, training of assessors) and a description of study instruments (eg, questionnaires, laboratory tests) along with their reliability and validity, if known. Reference to where data collection forms can be found, if not in the protocol	OK - Under Methods/Web-based study platform
	18b	Plans to promote participant retention and complete follow-up, including list of any outcome data to be collected for participants who discontinue or deviate from intervention protocols	Not applicable ("single shot" study)
Data management	19	Plans for data entry, coding, security, and storage, including any related processes to promote data quality (eg, double data entry; range checks for data values). Reference to where details of data management procedures can be found, if not in the protocol	OK - Under Methods/Web-based study platform
Statistical methods	20a	Statistical methods for analysing primary and secondary outcomes. Reference to where other details of the statistical analysis plan can be found, if not in the protocol	OK - Under Methods/Statistical Analysis
	20b	Methods for any additional analyses (eg, subgroup and adjusted analyses)	OK - Under Methods/Statistical Analysis
	20c	Definition of analysis population relating to protocol non-adherence (eg, as randomised analysis), and any statistical methods to handle missing data (eg, multiple imputation)	OK - Under Methods/Statistical Analysis - Imputation will not be required since only complete datasets will be analyzed
Methods: Monitoring			
Data monitoring	21a	Composition of data monitoring committee (DMC); summary of its role and reporting structure; statement of whether it is independent from the sponsor and competing interests; and reference to where further details about its charter can be found, if not in the protocol. Alternatively, an explanation of why a DMC is not needed	Not applicable ("single shot" study with no impact on participants' health)

	21b	Description of any interim analyses and stopping guidelines, including who will have access to these interim results and make the final decision to terminate the trial	Not applicable ("single shot" study with no impact on participants' health)
Harms	22	Plans for collecting, assessing, reporting, and managing solicited and spontaneously reported adverse events and other unintended effects of trial interventions or trial conduct	Not applicable given the study design
Auditing	23	Frequency and procedures for auditing trial conduct, if any, and whether the process will be independent from investigators and the sponsor	Not applicable ("single shot" study with no impact on participants' health)
Ethics and dissemination			
Research ethics approval	24	Plans for seeking research ethics committee/institutional review board (REC/IRB) approval	OK - Under Methods/Ethics Approval and Results
Protocol amendments	25	Plans for communicating important protocol modifications (eg, changes to eligibility criteria, outcomes, analyses) to relevant parties (eg, investigators, REC/IRBs, trial participants, trial registries, journals, regulators)	Not applicable ("single shot" study)
Consent or assent	26a	Who will obtain informed consent or assent from potential trial participants or authorised surrogates, and how (see Item 32)	OK - Under Methods/Consent and study sequence
	26b	Additional consent provisions for collection and use of participant data and biological specimens in ancillary studies, if applicable	Not applicable
Confidentiality	27	How personal information about potential and enrolled participants will be collected, shared, and maintained in order to protect confidentiality before, during, and after the trial	OK - Under Methods/Web-based study platform and in Multimedia Appendix 2 (info sheet)
Declaration of interests	28	Financial and other competing interests for principal investigators for the overall trial and each study site	OK - Under Statements/Funding and Statements/Conflicts of Interest
Access to data	29	Statement of who will have access to the final trial dataset, and disclosure of contractual agreements that limit such access for investigators	OK - Under Methods/Statistical Analysis
Ancillary and post-trial care	30	Provisions, if any, for ancillary and post-trial care, and for compensation to those who suffer harm from trial participation	Not applicable ("single shot" study with no impact on participants' health)
Dissemination policy	31a	Plans for investigators and sponsor to communicate trial results to participants, healthcare professionals, the public, and other relevant groups (eg, via publication, reporting in results databases, or other data sharing arrangements), including any publication restrictions	OK - Under Results
	31b	Authorship eligibility guidelines and any intended use of professional writers	Authorship clearly reported under "CRediT Author Statement" according to the CRediT guidelines.
	31c	Plans, if any, for granting public access to the full protocol, participant-level dataset, and statistical code	The protocol will be submitted for publication to JMIR Research Protocols, a peer-reviewed international open access journal
Appendices			
Informed consent materials	32	Model consent form and other related documentation given to participants and authorised surrogates	No consent form since consent will be gathered electronically - the platform will be accessible to peer reviewers.
Biological specimens	33	Plans for collection, laboratory evaluation, and storage of biological specimens for genetic or molecular analysis in the current trial and for future use in ancillary studies, if applicable	Not applicable
