# Supplementary material for: Impact of a Mobile App (LoAD Calc) on the Calculation of Maximum Safe Doses of Local Anesthetics: Protocol for a Randomized Controlled Trial
Source: JMIR Res Protoc. 2024 Jan 3;13:e53679. doi: 10.2196/53679 (PMC10794956; doi:10.2196/53679)
Supplement: Multimedia Appendix 2 [file resprot_v13i1e53679_app2.pdf]

Local anesthetics study - General information

Dear colleague,

We are currently conducting a study on the use of local anesthetics. Your participation would help us evaluate the methods used to determine the dosage of these agents.

Practical aspects

If you agree to participate, another anesthesiologist will replace you in the operating room for the duration of the study (about 1 hour). This replacement will be organized by the investigators. You will be asked to put your phone(s) on "flight" mode to avoid any disturbance during the study.

Data protection and privacy policy

Your identity (first and last names, date of birth) will not be recorded or even asked for. Only general demographic information will be collected, and it will be impossible to link your identity to your responses.

Frequently asked questions

Can I leave the study at any time?

Yes, you can leave the study at any time without justification.

Can I ask for my data to be deleted after the study?

No. As data cannot be linked to your identity, it will be impossible to delete specific data.

Who can I contact for further information?

You can contact :

- Mélanie Suppan - melanie.suppan@hcuge.ch
- Elias Fubini - elias.fubini@hcuge.ch
